# The Conservative Management of Choledocholithiasis With Ursodeoxycholic Acid

**DOI:** 10.7759/cureus.43850

**Published:** 2023-08-21

**Authors:** Daniel O Oluboyede, Mansoor Zafar, Farah Shirazi, Kevin Dsouza, Abdulmoen Abdulkarim, Kadir Hacikurt, Mark Whitehead

**Affiliations:** 1 Internal Medicine, Conquest Hospital, East Sussex Healthcare NHS Trust, St Leonards-on-Sea, GBR; 2 Gastroenterology, Hammersmith Hospital, Imperial College Healthcare NHS Trust, London, GBR; 3 Gastroenterology, Charing Cross Hospital, Imperial College Healthcare NHS Trust, London, GBR; 4 Medicine, Jinnah Medical & Dental College, Karachi, PAK; 5 Radiology, Conquest Hospital, East Sussex Healthcare NHS Trust, St Leonards-on-Sea, GBR; 6 Gastroenterology, Conquest Hospital, East Sussex Healthcare NHS Trust, St Leonards-on-Sea, GBR

**Keywords:** elderly patients, ercp, deranged liver function tests, frailty, ursodeoxycholic acid (udca), choledocholithiasis

## Abstract

Choledocholithiasis has been defined as the presence of stones within the common bile duct (CBD) with up to one-half of the cases remaining asymptomatic. We report a case of an 84-year-old frail male admitted for the treatment of pneumonia, pleural effusion, and bacteraemia with co-incidental deranged liver function tests (LFTs). Ensuing magnetic resonance cholangiopancreatography (MRCP) noted three CBD stones; however, the patient remained asymptomatic. After discussing the benefits and risks of treatment options with the gastroenterology team, the patient refused endoscopic retrograde cholangiopancreatography (ERCP) and opted for close monitoring in the community whilst taking ursodeoxycholic acid (UDCA). In the months following, his LFTs normalised, and repeat MRCP no longer showed stones. This case demonstrates that UDCA with close monitoring may be considered a non-invasive alternative treatment of CBD stones, particularly in elderly or frail patients with multiple comorbidities.

## Introduction

Choledocholithiasis has been defined as the presence of stones within the common bile duct (CBD). According to McNicoll et al., CBD stones are associated with 1-15% of cases of gall bladder stones (cholelithiasis) [1] and in a review by Costi et l., they found that cholelithiasis is associated with up to 20% of patients with CBD stone, with up to 50% of such cases being asymptomatic [2]. Furthermore, the updated guidelines of the British Society of Gastroenterology (BSG) on the management of common bile duct stones, suggest 10-20% of the patients with symptomatic gallstones have CBD stones [3].

Three different types of CBD stones have been reported: (i) cholesterol stones in obese patients or patients who have undergone weight loss, (ii) black pigment stones in patients who have had ileal resection, cirrhosis, and/or patients on total parenteral nutrition (TPN), and (iii) brown pigmented stones have been reported to be associated with a history of bacterial infections [4,5].

Endoscopic retrograde cholangiopancreatography (ERCP) to remove CBD stones followed by laparoscopic cholecystectomy remains the standard of care, especially in patients with cholangitis and/or a dilated biliary tree [6]. In less complicated cases, ERCP can be performed before, during, and after laparoscopic cholecystectomy [7,8]. However, we find a relative paucity of published current management plans for patients with multiple comorbidities and frailty, regarding alternate conservative options for patients who have CBD stones and are not fit for invasive procedures like ERCP. Here, we present a case of CBD stones successfully managed via a conservative approach.

## Case presentation

An 84-year-old male was referred to the gastroenterology team for clinic review for de-ranged liver function tests (LFTs) noted during his admission for pneumonia and bacteraemia. His comorbidities included diabetes mellitus type 2, atrial fibrillation, cardiac amyloidosis with global systolic impairment, decompensated heart failure with New York Heart Association (NYHA) functional classification score of 3, stable psoriasis and frailty. His Glasgow coma scale (GCS) score was 15/15, his abbreviated mental test score (AMTS) was 9/10, and his mini-mental state examination (MMSE) score was 27/30. His most recent echocardiogram (ECHO) showed mild concentric left ventricular hypertrophy, and globally severely impaired radial and longitudinal systolic function. The visual ejection fraction reported was 30-35% with dilated right ventricle, mild mitral regurgitation, and tricuspid regurgitation. His social history included mobility with a Zimmer frame, living with his wife, receiving assistance from carers once a day, and periodic reviewing by the community heart failure team. His medications included paracetamol (Tylenol) 0.5-1 g four times a day as needed, omeprazole 10 mg once a day (OD), bumetanide 2 mg twice daily, spironolactone 25 mg OD, dapagliflozin 10 mg OD, and apixaban 5 mg twice daily. He had a history of cough following ramipril use in the past.

After a one-week stay in the hospital, the patient was discharged following treatment of multi-lobar pneumonia with bilateral pleural effusions and streptococcal bacteraemia with intravenous Tazocin (piperacillin, tazobactam) 4.5 grams three times a day (TDS) for one week. With the resolution of symptoms and negative repeat blood cultures, he was discharged home on oral co-amoxiclav 625 mg TDS for another two weeks. His de-ranged LFT results persisted throughout his stay at the hospital (Table 1).

**Table 1:** Lab parameters on the day of admission and day 3 of admission

Lab parameter	Units of measurement	Lab results on admission	Lab results on day 3 of admission	Reference range
Serum sodium	mmol/L	132	134	133 - 146
Serum potassium	mmol/L	3.8	3.1	3.5 - 5.3
Serum urea	mmol/L	10	10.4	2.5 - 7.8
Serum creatinine	umol/L	106	100	59 - 104
Serum albumin	g/L	43	38	35-50
Serum alkaline phosphatase (ALP)	U/L	590	517	3-130
Serum alanine transaminase (ALT)	U/L	50	39	10-50
Serum bilirubin	umol/L	89	30	0-21
International normalized ratio (INR)	-	1.2	1.2	0.8-1.2
C- reactive protein (CRP)	mg/L	177	145	0-5
White cell count (WCC)	10^9^/L	16.23	8.5	4-11
Neutrophils		14.9	5.8	2-7.5
Plasma N-terminal-pro B-type natriuretic peptide (Plasma NT Pro BNP).	ng/L	8711	-	0-300

**Figure 1:**
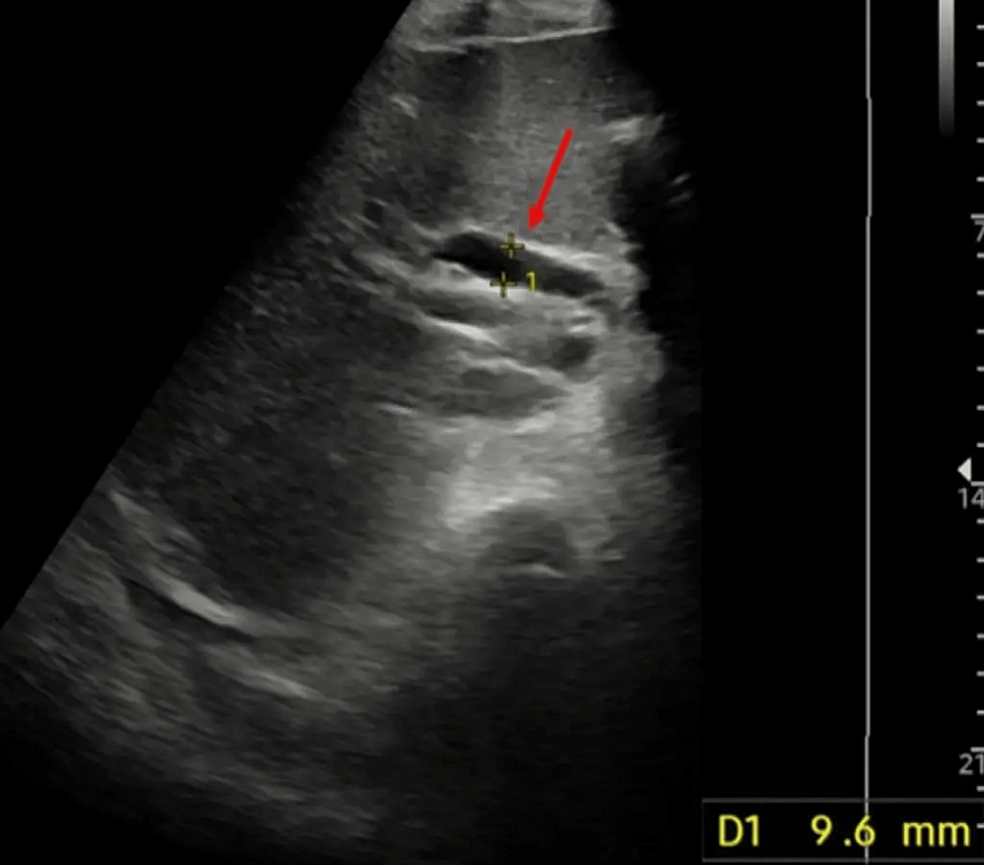
Ultrasound scan of the abdomen with axial grey-scale image showing slightly dilated common bile duct, 9.6 mm (red arrow)

Liver blood tests screening for possible hepatic, metabolic, and genetic aetiologies were inconclusive and an inpatient ultrasound scan (USS) of the abdomen showed a normal size liver with a normal ECHO pattern and no obvious focal lesions. However, the gallbladder was found to be grossly distended with sludge and debris within the lumen. The proximal common bile duct was seen to be dilated to 9.6 mm and the pancreas and distal duct were obscured by overlying bowel gas with no obvious intrahepatic tree dilatation seen (Figure 1). 

**Figure 2:**
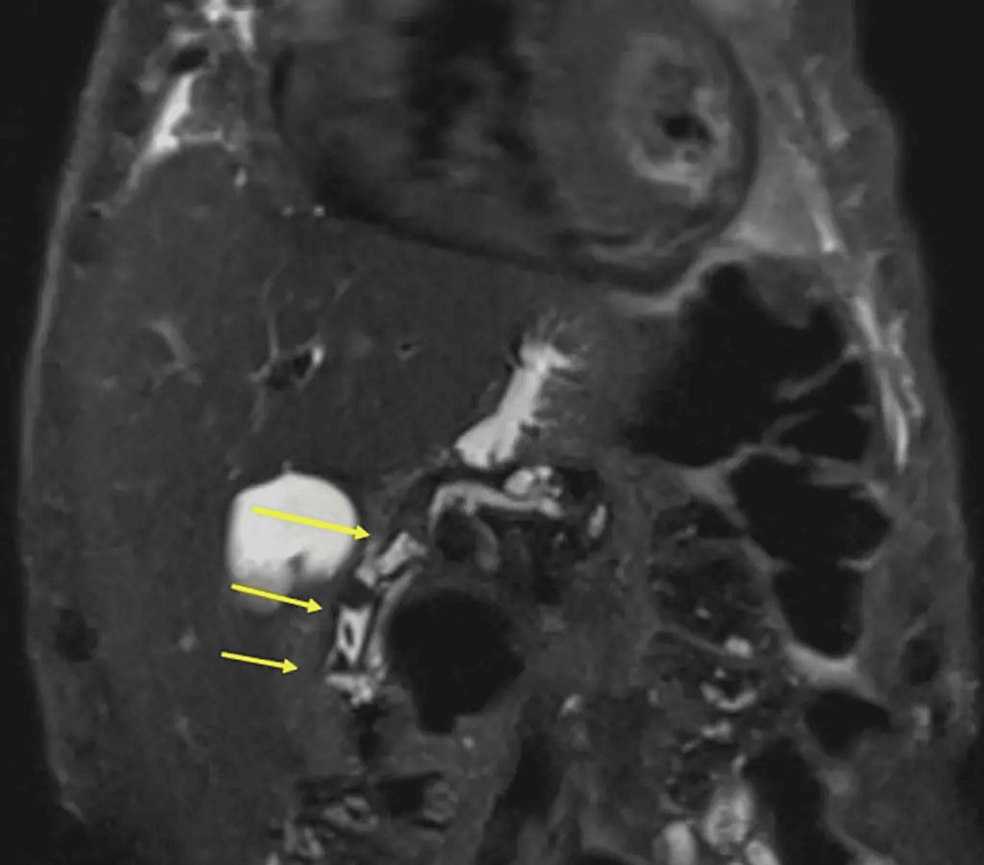
MRCP (coronal T2-weighted) HASTE image with fat saturation demonstrating three gallstones within the common bile duct (yellow arrows) MRCP: magnetic resonance cholangiopancreatography; HASTE: half fourier single-shot turbo spin-echo

Thereafter, an in-patient magnetic resonance cholangiopancreatography (MRCP) was requested that showed three CBD calculi with a normal calibre biliary tree and sludge within the gallbladder (Figure 2).

During the gastroenterology clinic review, four weeks post discharge from the hospital, the patient was noticed to have bilateral lower limb soft-pitting oedema to the knees and a jugular venous pulse visualised to be 3 cm above the sternal angle. Chest auscultation findings were coarse crackles at the right and left lung bases bilaterally, with pan-systolic murmur at a displaced heart apex that seemed to radiate to the ipsilateral axilla. His abdominal examination revealed no organomegaly, no tenderness, no fluid thrill, and he denied any right upper quadrant pain.

It was explained to him that CBD stones are associated with deranged LFTs with raised alkaline phosphatase (ALP) and he was given two options: (i) to proceed with an outpatient ERCP with sphincterotomy and bile duct clearance or (ii) the alternative suggested was conservative management with close observation, given the risks involved with proceeding with an ERCP in light of his comorbidities. During the clinic review, the benefits of ERCP were discussed, which would include reducing the risk of developing cholangitis in the future. The main risks were also discussed, including bleeding, side effects of sedation, pancreatitis, as well as rarer and more serious complications, including procedural failure.

**Figure 3:**
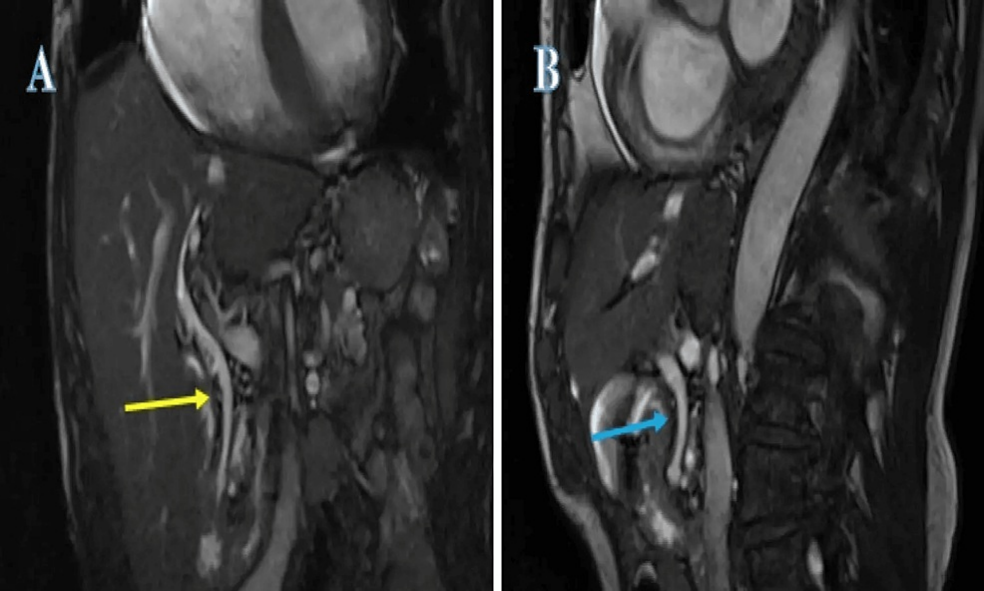
Repeat MRCP. (A) Coronal T2-weighted true fast imaging with steady-state free precession (TRUFI) image with fat saturation demonstrating normal calibre CBD free of stones (yellow arrow); (B) Coronal T2-weighted TRUFI image with fat saturation demonstrating normal calibre CBD free of stones (blue arrow)

After discussing the risks and benefits, the patient elected to go with the alternative option of conservative management. It was explained that if he developed significant right upper quadrant pain, fever, or felt very unwell, he should seek urgent medical attention. In the interim, the general practitioner (GP; family doctor) was advised to start the patient on ursodeoxycholic acid 300 mg twice a day and to give the patient a backup prescription of co-amoxiclav 625 mg TDS for one week. The patient was advised to take the ursodeoxycholic acid for three months, the continuation of which was to be decided during his next clinic review. The backup prescription of co-amoxiclav was intended for when the patient felt slightly unwell or developed mild abdominal pain but felt well enough that he did not think presenting to the hospital was necessary. He was booked for a follow-up clinic review in six months' time for clinical assessment and re-discussion of the options available with monthly interim LFT at the GP's surgery (clinic). His LFTs were normalised and a repeat MRCP at the gastroenterology clinic review showed the disappearance of the CBD stones (Figure 3).

The prescription for ursodeoxycholic acid was stopped with advice given to the GP for periodic LFT monitoring at the GP's surgery and the patient was discharged back to the care of the GP in the community.

## Discussion

The sensitivity of transabdominal ultrasound scans in the detection of gallbladder stones has been reported to be up to 96%. This sensitivity to detect CBD stones drops to 50% or less. In most cases, this is owing to bowel gas patterns obscuring the visibility [9]. In such cases, reliance is on indirect signs including CBD dilatation, although this is contentious as the size of CBD varies between 5 mm and 11 mm with an increase in size associated with advancing age and/or post-cholecystectomy [10]. Another helpful indirect sign is multiple and small-sized stones from the gallbladder, which are more likely to move distally to the CBD [11]. Magnetic resonance cholangiogram (MRC) or MRCP is considered the gold standard investigation for CBD stone detection with a sensitivity of 85-92% and specificity of 93-97% [12]. In patients with CBD stones, the standard of care is ERCP, which is both diagnostic and therapeutic, followed by cholecystectomy in patients with co-existent gallbladder stones [13].

Untreated asymptomatic CBD stones have been reported to be associated with complications including cholangitis and/or pancreatitis and could potentially be life-threatening [14]. However, Saito et al. have been the first to outline a retrospective multicenter study of 425 patients undergoing ERCP for asymptomatic and symptomatic CBD stones. They have reported complications post-ERCP to be 26.9 % in asymptomatic patients with CBD stones as opposed to 3.3% in symptomatic patients. They have also concluded complications were more severe in elderly patients [15]. Although current guidelines still recommend offering ERCP to patients with asymptomatic CBD stones, the evidence remains of low quality [16].

The use of bear bile, which is extracted from black adult bears, in Chinese Traditional Medicine dates back to the Tang dynasty. Hammarsten, a Swedish research worker, first identified ursodeoxycholic acid from polar bear bile in 1902; it is said that he ran out of sample and could not complete the crystallization. Shoda from Okayama University successfully crystallised it in 1927 and named it ursodeoxycholic acid (“Urso”, bear in Latin). Makino et al. were the first to show the dissolution of cholesterol gallstones with UDCA in Japan [17]. This resulted in the global use of UDCA for the dissolution of gallstones. However, it also has a significant reported role in primary biliary cirrhosis, colon cancer prophylaxis and prevention of adenoma recurrence via antiproliferative effects, immune modulation in patients suffering from AIDS, and, lastly, it may have potential as a cell membrane stabiliser in central nervous system pathologies due to its ability to cross the blood-brain barrier [18].

For patients with large and multiple CBD stones, Hormati et al. have recommended UDCA use along with CBD stenting, or prior use, to assist with reducing the stone size and facilitating the extraction of the CBD stone [19]. Roda et al. conducted a prospective clinical trial on 223 patients using CDCA and its 7β isomer UDCA in patients with gallstones. They found UDCA more efficacious and associated with a faster response without causing untoward side effects of diarrhoea and/or transaminitis [20].

In the past, Salvioli et al. had conducted a prospective clinical trial of 24 patients divided into a UDCA treatment group and a placebo group in 1983 and showed seven patients in the UDCA treatment group had complete disappearance of the CBD stones and one patient had partial resolution [21]. Johnson et al. in 1993 published outcomes for 22 patients post unsuccessful sphincterotomies with stents placed in which 10 patients were assigned to the UDCA treatment group and the remaining 12 to the control group [22]. Nine of 10 patients in the UDCA treatment group had complete stone clearance, with 41 of 42 stones removed during a follow-up period of 9±2 months. On the contrary, among the control group, no patients had complete clearance and only six of the 40 stones were removed after a follow-up period of 31±6 months.

Guarino et al., in 2013, suggested that UDCA remains the cheapest, safest and best-tested drug for gallstone disease [23]. They concluded that the story would not end there but deserved further attention and investigation. We suggest a step ahead with the recommendation of UDCA as being the cheapest, safest and best-tested drug in the treatment of CBD stones, particularly in patients who are frail with comorbidities that preclude invasive procedures, thus ensuring better outcomes in such patients.

Lastly, Ryuichi Yamamoto et al., in 2016, conducted a multi-centre randomized trial comparing the rates of CBD stones after CBD stone removal among patients given UDCA versus patients not receiving it [24]. The primary endpoint was labelled the recurrence rate of the CBD stones. They noticed the recurrence rate of CBD stones to be 6.6% in the UDCA group and 18.6% in the untreated group (p = 0.171) [24]. This practically could suggest that the use of UDCA would be potentially beneficial in patients who are frail and considered high risk for invasive procedures as in our patient. Thus, the story outlined by Guarino et al. [23] continues to evolve, advocating further attention and investigations.

## Conclusions

Current guidelines recommend offering ERCP to patients with asymptomatic CBD stones despite the lack of robust evidence in support of this position and an association with increased complications compared to their symptomatic counterparts, particularly in the frail population. This case demonstrates that UDCA may be considered a non-invasive alternative treatment of CBD stones in elderly or frail patients with multiple comorbidities.
